# Characteristics of Soil and Organic Carbon Loss Induced by Water Erosion on the Loess Plateau in China

**DOI:** 10.1371/journal.pone.0154591

**Published:** 2016-04-28

**Authors:** Zhongwu Li, Xiaodong Nie, Xiaofeng Chang, Lin Liu, Liying Sun

**Affiliations:** 1 State Key Laboratory of Soil Erosion and Dryland Farming on the Loess Plateau, Institute of Soil and Water Conservation, CAS and MWR, Yangling, Shaanxi Province, 712100, PR China; 2 College of Environmental Science and Engineering, Hunan University, Changsha, 410082, PR China; 3 Key Laboratory of Environmental Biology and Pollution Control (Hunan University), Ministry of Education, Changsha, PR China; 4 Key Laboratory of Water Cycle and Related Land Surface Processes, Institute of Geographic Sciences and Natural Resources Research, Chinese Academy of Sciences, Beijing, 100101, PR China; Beijing Normal University, CHINA

## Abstract

Soil erosion has been a common environmental problem in the Loess Plateau in China. This study aims to better understand the losses of soil organic carbon (SOC) induced by water erosion. Laboratory-simulated rainfall experiments were conducted to investigate the characteristics of SOC loss induced by water erosion. The applied treatments included two rainfall intensities (90 and 120 mm h^-1^), four slope gradients (10°, 15°, 20°, and 25°), and two typical soil types- silty clay loam and silty loam. Results showed that the sediment OC enrichment ratios (ERoc) in all the events were relative stable with values ranged from 0.85 to1.21 and 0.64 to 1.52 and mean values of 0.98 and 1.01 for silty clay loam and silty loam, respectively. Similar to the ERoc, the proportions of different sized particles in sediment showed tiny variations during erosion processes. No significant correlation was observed between ERoc values and the proportions of sediment particles. Slope, rainfall intensity and soil type almost had no impact on ERoc. These results indicate that the transportation of SOC during erosion processes was nonselective. While the mean SOC loss rates for the events of silty clay loam and silty loam were 0.30 and 0.08 g m^-2^ min^-1^, respectively. Greater differences in SOC loss rates were found in events among different soil types. Meanwhile, significant correlations between SOC loss and soil loss for all the events were observed. These results indicated that the amount of SOC loss was influenced primarily by soil loss and the SOC content of the original soil. Erosion pattern and original SOC content are two main factors by which different soils can influence SOC loss. It seems that soil type has a greater impact on SOC loss than rainfall characteristics on the Loess Plateau of China. However, more kinds of soils should be further studied due to the special formation processes in the Loess Plateau.

## Introduction

Soil erosion has become a globally prevalent problem that threatens the ecological environmental quality as well as the sustainable development of economic and society [1, 2]. It not only leads to soil and water loss but also causes the transport of soil organic carbon (SOC) [3, 4]. Conventional studies on soil erosion include assessing the damage to soil and crop yield as well as investigating the off-site effects of soil erosion on water quality, runoff, and sediment loads in rivers; while in recent years, the role of soil erosion in global geochemical cycles has been gaining considerable attention [1, 5]. For this reason, the movement of SOC during erosion processes should be elucidated.

SOC is lost in two forms, sediment-bound organic carbon and dissolved organic carbon, with the former (60%–90%) often being the dominant mechanism [6, 7]. Polyakov and Lal [3] found that SOC loss occurred through increased erosion-induced mineralization and preferential transportation by runoff. Studies showed that the removal of SOC undergoes four stages: dispersion by raindrop and runoff, transport by runoff, redistribution in landscape or being deposited in micro-depression, and transport to the outlet [8, 9]. SOC loss is influenced by numerous factors, such as rainfall intensity, slope gradient, tillage, and soil type. Among these factors, rainfall intensity and slope gradient are the most extensively studied [10–13]. SOC loss is widely believed to increase as the rainfall intensity increases. Although lost SOC is not significantly correlated to runoff directly [7, 14], higher rainfall intensity leads to a decrease in infiltration and an increase in runoff, thereby providing additional energy for the motion of sediment and SOC [7]. Meanwhile, rainfall intensity also affects soil detachment by raindrop impact and detached particles transportation by runoff [15]. For slope gradient, study results have shown that the SOC loss influenced by slope gradient presented no clear trend, and that rainfall intensity had a larger effect on sediment yield than slope [10]. In fact, these factors mainly affect SOC loss by influencing overland flow and rill and interrill erosion processes [16].

Erosion is a selective process in transporting soil materials, and SOC is often transported preferentially during erosion processes [17]. The preferentially migration of SOC is often expressed as an enrichment ratio (ER) [18]. The ER of organic carbon (ERoc) reflects the migration characteristics of SOC in erosion processes and is also related to as numerous factors. Simulated rainfall experiments have shown that the ERoc increased with slope length and decreased with rainfall duration [3]. Martinez-Mena et al. [19] indicated that the close rainfall characteristics mainly related to SOC mobilization are rainfall intensity and rainfall duration. During low-intensity rainfall events, ERoc in the sediment was higher than that during high-intensity storms [7, 20, 21]. The reason is that more coarse sediments, which have a low content of SOC, were transported in high-intensity storms, thereby diluting the SOC concentration of the sediment. With regard to rainfall duration, researchers have shown that the sediment would become less enriched in carbon as time passes during an event because more carbon-rich fine aggregates were depleted early in the event [3, 19, 22, 23]. The non-homogeneous distribution of organic carbon among particles of various sizes (i.e. clay and fine silt) is often considered the reason for the high ERoc values in sediment [24, 25]. Sediment particle distribution and SOC content were influenced by runoff and raindrop peeling, which are also influenced by the rainfall characteristics. However, the extent to which rainfall characteristics are responsible for this enrichment mechanism is still unclear [19]. Therefore, further studies should be conducted to detect the impact of rainfall processes on SOC dynamics and consequently clarify the SOC loss during water erosion.

As an important carbon pool, the Loess Plateau of China is a region subjected to severe soil erosion, especially in hilly regions [26]. Numerous field and laboratory studies have been conducted to investigate the effects of soil erosion on the ecological environment. However, the majority of these studies were focused on the soil loss and migration characteristics of the sediment during erosion processes [27–30], and limited attention have been given to the loss of SOC. Therefore, the present study aims to reveal the features of SOC loss during erosion processes in the Loess Plateau. Specifically, two typical soils of the Loess Plateau were selected to analyze the characteristics of SOC loss. Simulated rainfall experiments with different rainfall intensities, slope gradients, and soil types were conducted. In addition, the soil and SOC loss rates, as well as the selective migration of both SOC and sediment particles, were examined. The results of this study can aid in improving the management of land and water resources and promoting the storage of organic carbon.

## Materials and Methods

### Ethics Statement

In this study, soil sampling and sample determinations conducted were permitted by the local authorities (local farmers). We also obtained a permission from the local authorities (local farmers) for reporting research results to the public. And no other specific permissions were required for these activities. Because the further study after soil sampling were conducted in laboratory. In addition, the laboratory studies did not involve endangered or protected species.

### Simulated rainfall experiments

**Fig 1:**
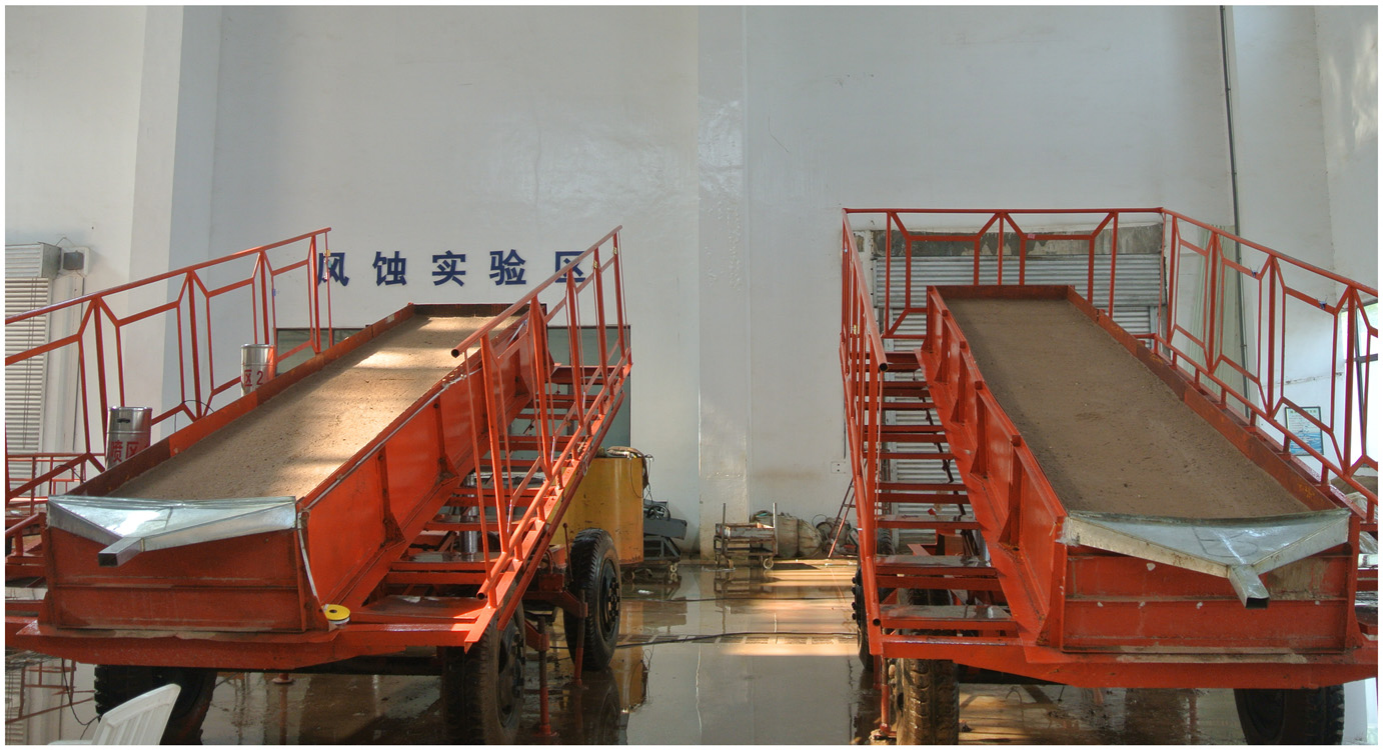
Soil plots of the simulated rainfall experiments.

Simulated rainfall experiments were conducted in the state key laboratory of soil erosion and dryland farming on the Loess Plateau. The characteristics of the rainfall simulator were well described by Pan and Shangguan [31]. Rainfall intensities were adjusted through nozzle sizes and water pressure. Experimental plots were constructed with metal sheets, and the dimensions were 5 m (length) × 1 m (width) × 0.5 m (depth). The plots were placed on movable platforms for ease of movement around. The plot was adjusted electrically to a desired slope between 0° and 30° (Fig 1). Calibrations of rainfall intensities were conducted prior to the experiments.

The soils used in these experiments were silty clay loam from Changwu County (35°12′ N, 107°47′ E) and silty loam from Suide County (37°31′ N, 110°16′E), Shaanxi Province, China. The two soils were distributed in southern and northern parts of the Shaanxi Province, respectively. Both of the soils have suffered serious erosion, and field monitoring stations on water erosion have been established on these two sites. They represent two typical soils on the Chinese Loess Plateau. The soils were collected from farmlands at a depth of 20 cm. No organic fertilizer was applied to the farmlands. The basic physicochemical properties of the soils can be obtained from Table 1.

**Table 1:** Characteristics of the study soils.

Soil type	SOC (g kg-1)	CEC (cmol kg^-1^)	pH	Bulk density (g cm^-3^)	Sand (%)	Silt (%)	Clay (%)
Silty clay loam	7.8±0.43	12.4±0.72	8.3±0.22	1.20±0.14	9.5±0.8	69.3±2.1	21.2±2.9
Silty loam	2.7±0.23	8.1±0.18	8.7±0.47	1.25±0.08	32.1±3.5	55.8±3.9	12.1±0.4

The collected soils were air-dried to a moisture content of about 10%, and plant residuals were removed. Then, the samples were gently crushed and passed through a 10 mm sieve. The used soils were sampled and controlled to constant moisture before being used in the experiments. To obtain a better drainage, a 20 cm layer of sand was placed at the bottom of a plot. The remaining 30 cm of the plot was divided into three 10 cm layers, and each layer was packed with thoroughly mixed soils. The bulk density of 1.20 and 1.25 g cm^-3^ were obtained for silty clay loam and silty loam, respectively. These bulk densities corresponded to the local values of the farmland. The upper soil layer was raked lightly before the rainfall experiments to diminish discontinuity.

For rainfall simulations, rainfall intensities of 90 and 120 mm h^-1^, which corresponded to the typical rainfall intensity in sub-humid climate regions of China were used in the experiments. In addition, the topographical feature of the Chinese Loess Plateau is complicated, and 25° slope is the maximum slope for cultivated land according to the Chinese Soil and Water Conservation Act [29]. Therefore, the slopes of 10°, 15°, 20°, and 25° were applied. Overall, the rainfalls include two soils, two rainfall intensities, and four slope gradients. Considering that experiments were conducted in two replicates, 32 simulated rainfalls were being conducted in total. For different intensity rainfalls, the total rainwater was designed to be of the same value. Therefore, rainfalls of 90 and 120 mm h^-1^ lasted for 60 and 45 min, respectively.

Once the rainfall started, the rainfall time and the surface runoff start time were recorded. Runoff with sediment was collected in a bucket at 3 min intervals. The total runoff volumes were also measured and recorded at the same time. Then, the collected mud samples were deposited, separated from the water, dried in a forced-air oven at 60°C until a constant mass was achieved and weighed for determining the sediment content. Then, the sediment samples were stored for the measurement of SOC concentration.

Another sample of runoff with sediment was collected in a beaker at 3 min intervals during rainfall. The collected samples were immediately used to analyze the effective particle size distribution (PSD) with the use of a Malvern Mastersizer 2000 laser diffraction device (Malvern Instruments Ltd., UK). After the PSD was determined, the sediment samples were treated with hydrogen peroxide to remove organic matter, dispersed in sodium hexametaphosphate, and then subjected to ultrasonic dispersion to obtain the ultimate PSD of the sediment using the Malvern Mastersizer 2000 laser diffraction device.

The organic carbon concentrations of soils and sediments were determined by applying the dichromate oxidation method proposed by Walkley and Black [32]. The ERoc of sediments was calculated as follows:

ERoc=SOCsedimentSOCplot  soil (1)

where SOC _sediment_ is the organic carbon content (g kg^-1^) in sediments and SOC _plot soil_ is the organic carbon content (g kg^-1^) in plot soils.

### Statistical analyses

Statistical analysis was performed using SPSS 20.0 for Windows. The values of SOC, ERoc, PSD, and so on were the mean values of the two replicate events. Spearman correlation was used to evaluate the association between SOC loss rate and soil loss rate. A simple linear correlation was used to determine if ERoc and PSD were related. Significant differences in sediment and runoff loss rate between events with different slope gradients were analyzed by one-way ANOVA.

## Results

### Rainfall and sediment loss properties

The time to start runoff was 83–170.5 s and 235–470 s for the events of silty clay loam and siltly loam, respectively (Table 2). The rainfalls of siltly loam had a longer time to start runoff than rainfalls of silty clay loam. Runoff rates for events of silty clay loam and siltly loam were generally in the range of 1.20–1.91 mm min^-1^ and 0.43–1.47 mm min^-1^, respectively. The effect of slope gradient on runoff rate did not demonstrate any clear trend. Average sediment loss rates for events of silty clay loam varied between 20.59 and 64.18 g m^-2^ min^-1^, whereas the values for events of silty loam varied in 4.65–121.37 g m^-2^ min^-1^. For all the events of silty clay loam and siltly loam, sediment yield rate increased with rainfall intensity and slope gradient. During low rainfall intensity, sediment loss rate for events of silty loam was lower than that for events of silty clay loam; however, an opposite scenario occurred during high rainfall intensity. The total amount of the lost soil and SOC, which were similar to the average loss rate, also increased with slope gradient and rainfall intensity. Significant differences in total loss amount of soil and SOC were observed between rainfall events with slopes of 10° and 25°.

**Table 2:** Summary parameters for each rainfall experiment.

Soil type	Rainfall intensity (mm h^-1^)	Slope gradient (°)	Time to start runoff (s)	Rill erosion devleopment	Average runoff rate (mm min^-1^)	Sediment yield rate (g m^-2^ min^-1^)	Total soil loss (kg m^-2^)	Total SOC loss (g m^-2^)
Silty clay loam	90	10°	149.5a	N	1.20a	20.59b	1.18b	9.58b
		15°	163a	N	1.24a	22.23b	1.27b	9.61b
		20°	137a	N	1.22a	24.76b	1.41b	10.60b
		25°	170.5a	Y	1.24a	51.68a	2.97a	22.53a
	120	10°	126.5a	N	1.81b	29.18b	1.25b	9.88b
		15°	126a	N	1.80b	48.93ab	2.05ab	15.98ab
		20°	83b	Y	1.71c	61.98a	2.62a	19.61a
		25°	92b	Y	1.91a	64.18a	2.79a	21.71a
Silty loam	90	10°	356b	N	0.75a	4.65c	0.25b	0.62b
		15°	243c	Y	0.69ab	7.48b	0.38ab	1.65a
		20°	390b	Y	0.60bc	9.56a	0.52a	1.12ab
		25°	470a	Y	0.43c	5.16c	0.25b	0.61b
	120	10°	235a	N	1.47a	13.17b	0.55c	1.23c
		15°	242a	Y	1.17a	82.49ab	3.47b	7.18ab
		20°	246a	Y	1.16a	101.87a	4.02ab	8.48a
		25°	287.5a	Y	0.62b	121.37a	5.10a	9.53a

N (Y) in the table means that rill erosion was (was not) found in the event. Numbers followed by a different lower case letter within the same column are significantly different (p < 0.05) with slope gradient.

**Fig 2:**
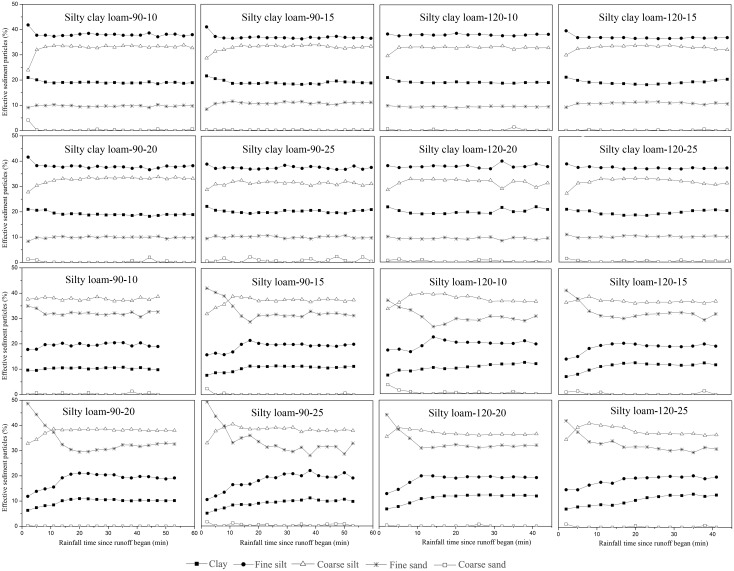
Temporal variations in the percentage of ultimate sediment particles under different rainfall events. Silty clay loam and Silty loam represent the soils, 90 and 120 represent the rainfall intensities (mm h^-1^), and 10, 15, 20 and 25 represent the slope gradient (°).

Sediment sizes were classified as clay-size (<0.002 mm), fine silt-size (0.002–0.02 mm), coarse silt-size (0.02–0.05 mm), fine sand-size (0.05–0.25 mm), and coarse sand-size (>0.25 mm). For silty clay loam, the ultimate PSD of sediment in all events followed the order of fine silt > coarse silt > clay > fine sand > coarse sand (Fig 2). The percentages of the coarse sand can be almost neglected. Except for a slight change at the initial time since runoff, the variation of particle distribution was almost constant with rainfall duration. Thus, rainfall processes do not seem to have any impact on the sediment particles transportation. For events of silty loam, the sediment particle sizes in all rainfall events were varied in the order of coarse silt > fine sand > fine silt > clay > coarse sand (Fig 2). Compared with sediment particles of the events of silty clay loam, relatively larger changes in particles, especially for the sand particles, at the initial of rainfall events were observed. In general, the effects of erosion processes only had a small impact on the ultimate sediment particle distribution. By contrast, soil type largely influences on sediment PSD.

### ERoc in sediment

**Fig 3:**
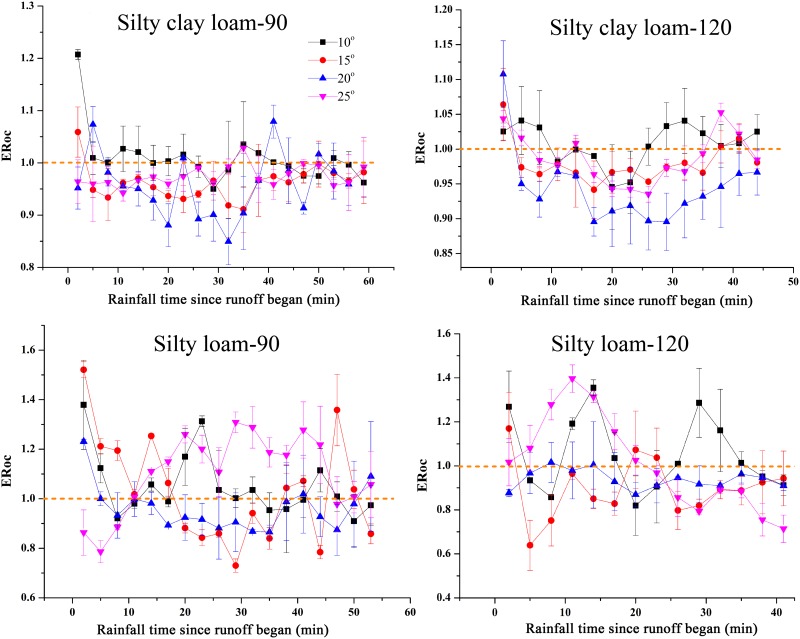
Enrichment ratio of organic carbon (ERoc) in sediment for different rainfall events. The error bars denote the standard deviations. Silty clay loam and Silty loam represent the soils, 90 and 120 represent the rainfall intensities (mm h^-1^).

ERoc in sediment for different rainfall events are presented in Fig 3. The ERoc values in events of silty clay loam-90 and silty clay loam-120 varied in the ranges of 0.85–1.21 and 0.90–1.11, respectively. For the events of silty loam-90 and silty clay loam-120, the ERoc values varied in the ranges of 0.73–1.52 and 0.64–1.40, respectively. Obviously, all of these values varied around 1, and the ERoc values exhibited no systematic change with rainfall time. Similar ERoc curves were found among events that had the same rainfall intensity. For both soils, the ERoc in the sediment decreased at the initial time after the runoff began. Linear correlation analysis showed that the correlations between ERoc and different sizes of sediment particles varied with rainfall event and soil type (Table 3). Most of the correlations were insignificant (*P*>0.05), and the results indicated that the ERoc did not correlate with the sediment particles. These findings suggest that no uniform rule can be established for the correlations between the rainfall events. Notably, significant positive correlation between ERoc and clay cannot be found in all of the events.

**Table 3:** Correlation analysis between ERoc and different size particles.

Soil type	Rainfall intensity (mm h^-1^)	Slope gradient (°)	Non-despised soil particles					Dispersed sediment particles		
			<0.002	0.002–0.02	0.02–0.05	0.05–0.25	>0.25	Clay	Silt	Sand
Silty clay loam	90	10°	0.23	-0.05	0.04	0.04	-0.06	-0.06	-0.14	0.09
		15°	0.31	0.25	-0.30	-0.19	-0.37	-0.37	-0.16	-0.23
		20°	-0.03	0.03	-0.11	-0.08	0.43	0.43	-0.34	0.29
		25°	-0.47	-0.46	0.04	0.09	0.46	0.46	-0.25	0.39
	120	10°	0.05	-0.50	0.01	0.60*	0.08	0.08	-0.52*	0.43
		15°	0.28	-0.33	-0.52*	-0.47	0.23	0.23	-0.53*	-0.54*
		20°	0.40	0.24	-0.37	-0.18	0.08	0.08	-0.34	0.03
		25°	0.83**	0.48	-0.84**	-0.08	0.24	0.24	-0.66**	0.07
Silty loam	90	10°	0.03	0.08	-0.25	-0.08	0.26	0.26	-0.12	0.07
		15°	-0.64**	-0.54*	-0.16	0.47*	0.40	0.40	-0.42	0.48*
		20°	-0.60**	-0.59**	-0.28	0.54*	0.40	0.40	-0.53*	0.54*
		25°	0.52*	0.64**	0.21	-0.59*	-0.31	-0.31	0.60**	-0.62**
	120	10°	-0.02	0.13	0.03	-0.13	-0.16	-0.16	0.16	-0.10
		15°	0.17	0.16	-0.63*	-0.24	0.14	0.14	-0.09	-0.15
		20°	-0.18	0.29	0.55*	-0.33	-0.23	-0.23	0.36	-0.32
		25°	-0.77**	-0.68**	0.81**	0.71**	-0.16	-0.16	0.50	0.65*

** Correlation is significant at the 0.01 level (two-tailed),

*correlation is significant at the 0.05 level.

### SOC loss

**Fig 4:**
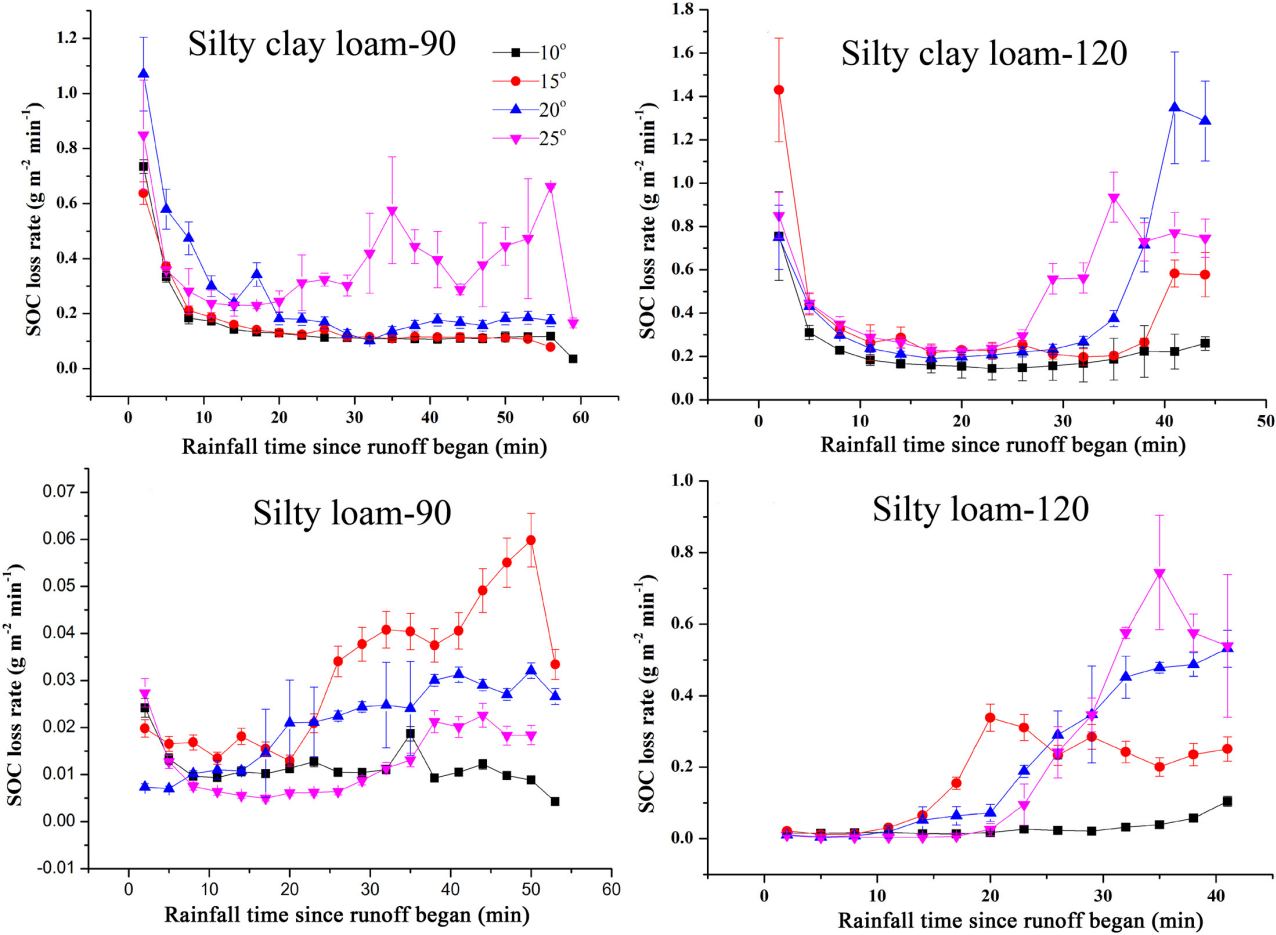
Temporal variations of SOC loss rate for different rainfall events. The error bars denote the standard deviations. Silty clay loam-90 and Silty clay loam-120 (Silty loam-90 and Silty loam-120) denote the rainfall events of Silty clay loam (Silty loam) soil with rainfall intensities of 90 mm h^-1^ and 120 mm h^-1^.

**Fig 5:**
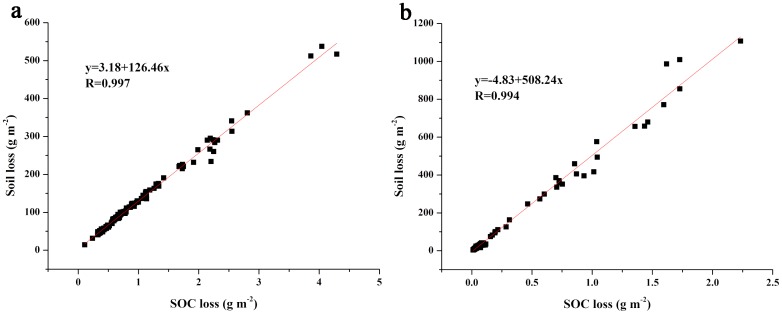
Relationships between SOC loss and erosion intensity in (a) events of silty clay loam and (b) events of silty loam.

The SOC loss rates presented differences among rainfall events (Fig 4). For events of silty clay loam, the SOC loss rates in 90 and 120 mm h^-1^ rainfall events varied in the range of 0.08–1.07 g m^-2^ min^-1^ and 0.14–1.43 g m^-2^ min^-1^, respectively. In all these events, the values initially decreased, then remained constant, and in some events, eventually increased again. For events of silty loam, the SOC loss rates varied in the range of 0.0042–0.21 g m^-2^ min^-1^ and 0.0036–0.74 g m^-2^ min^-1^ for 90 and 120 mm h^-1^ rainfall events, respectively. For all the events, the values of SOC loss rate varied with rainfall intensity and soil type. Three trends of SOC loss rate were observed: decreasing–constant–increasing, constant–increasing, and constant. Nevertheless, there was no obvious regularity in the effect of slope on SOC loss rate. While, the mean loss rates and total amounts of SOC in the events of the same soil increased as rainfall intensity and slope gradient increased. These findings suggest that SOC loss was significantly affected by erosion processes. Significant linear relationships between SOC loss and erosion intensity (soil loss) were found in the events of silty clay loam and silty loam, and the correlation coefficients reached more than 0.99 (Fig 5). The SOC loss increased with an increase in erosion intensity, which is a function of the amount of soil loss. Eroded soils played an important role in determining the loss of SOC during erosion processes. However, for the events of different soils, the SOC loss and soil loss was not directly proportional. The SOC content in original soil is another important way by which soil type can influence SOC loss.

## Discussion

### Selective transportation of organic carbon in sediment

In this study, the results showed that the ERoc in the sediment varied in the range of 0.85–1.21 and 0.64–1.52 for events of silty clay loam and silty loam, respectively (Fig 3). Most of the values varied between 0.8 and 1.2, indicating that the SOC cannot be transported preferentially during water erosion. This result is inconsistent with the findings of most previous studies. For example, Jia et al. [33] conducted an experiment to monitor the SOC loss on the Loess Plateau and found that the ERoc ranged between 1.58 and 4.12. Jin et al. [34] conducted rainfall experiments on the Loess Plateau and found that the ERoc in the sediment varied from 1.01 to 2.24 and remaining ≥ 1. Furthermore, the ERoc values in this study were also lower than the values that were reported in literatures, such as 1.0–1.7 [3], 0.9–2.6 [20], and 1.3–4.1 [35]. In addition to the effects of soil type and erosion conditions (indoor (field) simulations or natural rainfalls), many factors have been considered related to ERoc values. In general, the enrichment of organic carbon in sediment is often attributed to the preferential transportation of: (a) poorly decomposed noncohesive plant fragments [36]; (b) fine-sized sediments that are richer in silt and clay particles and SOC [19]. However, the study soils contained very few plant residues because they were collected from croplands, and the crop straw was harvested in the fall and the weeds were controlled to increase crop yield. Less plant residues may be one of the most critical influences factors on the lower ERoc values when compared to previous studies. In addition to the lower values, in this study, the variation trends of ERoc varied with soil types but were almost not affected by rainfall intensity and slope gradient (Fig 3). In contrast to our findings, researches have shown that ERoc tended to decrease with slope gradient [33] and rainfall intensity [35]. The selectivity of SOC has been partly attributed to the transported of fine-sized sediments [19] that are considered to be affected by rainfall intensity, soil type, and slope gradient [29, 30]. However, no clear correlation was found between ERoc and sediment particles in this study (Table 3), and finer particles were not found to be transported preferentially (Fig 2). Shi et al. [29] concluded that the PSD in sediment cannot be influenced by the PSD of the original soil and the breakdown and settling velocity of different size classes aggregate during erosion. However, the PSD was almost constant for all rainfall events (Fig 2). Although with different erosion processes, rainfall events with the same soil had similar PSD. The PSD of the original soil was believed to be the main influencing factor. The blowing and carrying of wind was mainly responsible for the formation of the Loess Plateau [37]. Moreover, a selective process of soil particles occurred during the soil formation. As a consequence, the PSD of the soil of the Loess Plateau may hamper the selective transportation of soil particles and influence the ERoc in the sediment. Therefore, the SOC source and the broken degree of aggregates may be the causes of the lower and constant ERoc values in this study. In addition, the selective transportation of SOC was thought to be affected by rainfall intensity, and a previous study found that the ERoc in the sediment during low-intensity storms was higher than that during high-intensity storms [21]. In this study, the applied rainfall intensities (90 and 120 mm h^-1^), which were thought to be high enough to transport more coarser and heavier particles, diluted the SOC concentration in the sediment and decreased the ERoc [7].

### SOC loss

During water erosion, the lost SOC was mainly in particulate phase [6, 7]. Starr et al. [38] suggested that soil loss can be multiplied by the percentage of SOC in the near-surface soil and an enrichment ratio to obtain SOC loss. And SOC losses mainly depend on the ERoc of the sediment, the SOC content of original soil, and the total soil loss. As reported in literatures [3, 11–14, 20], soil loss and ERoc were affected by numerous factors, such as rainfall intensity, slope gradient, soil type, and rainfall duration. However, the study showed that the ERoc showed relatively stable values, which may provide convenience for the calculation of the lost SOC during water erosion. The variations of the total lost amount of SOC were highly related to that of the total lost amount of soil (Table 2). Significant linear correlations between SOC loss and soil loss were observed in the events of silty clay loam and silty loam (Fig 5), indicating that the SOC loss was closely associated with soil loss. Consequently, the SOC loss, which differed from the ERoc, was strongly influenced by erosion processes. Rainfall intensity, slope gradient, soil type, rainfall duration, tillage, and residues cover are important factors that affect runoff generation and soil loss [15, 16, 39–42]. Similar to previous study [10], the results showed that the average soil loss rate increased as rainfall intensity and slope gradient increased, and rainfall intensity had a larger effect on sediment yield than slope gradient. Furthermore, the sediment loss rates for events of silty loam were lower than that for events of silty clay loam in low-intensity rainfalls, but the opposite occurred in high-intensity rainfalls. These results might be attributed to the sediment erosion pattern that was affected by runoff generation [43]. The time to start runoff in rainfall of silty loam events was longer than that in events of silty clay loam (Table 2), and huge differences were found among the events of different soils. Based on the simulated rainfall results, it is concluded that the surface runoff in rainfalls of silty clay loam were generated by the infiltration excess (Horton) mechanisms and in rainfalls of silty loam were generated by the saturation excess (Dunn) mechanisms. Under this situation, sheet erosion was the main manner of soil loss for events of silty clay loam and events of silty loam with low-intensity rainfall, while rill erosion was the main manner for events of silty loam in high-intensity rainfall. Erosion patterns have a profound impact on sediment yield [10, 44]. In addition, the original SOC content may also be an important factor that influences SOC loss. Although more soil was lost in silty loam-120 events than in silty clay loam-120 events, the SOC loss was higher in events of silty clay loam-120 than that in silty loam-120 events. The higher OC content in original soil was believed to be the main reason. Therefore, soil types, which affect the SOC loss through erosion pattern and original OC content, should be paid more attention in the study of SOC loss in the Loess Plateau. In consideration of the formation processes of the soil on the Loess Plateau, the characteristic of zonality in soil and SOC loss might be found. Thus, further study should be conducted in the future.

## Conclusions

Soil erosion transported plenty of sediment and associated organic carbon in the Loess Plateau of China. However, different from previous studies, the SOC as well as soil particles in the sediment did not show selective transportation during erosion processes, which will provide convenience for the calculation of the lost SOC during water erosion. And significant linear relationships were observed between SOC loss and sediment loss. This result indicated that the loss amount of SOC in the Loess Plateau of China could be well predicted by transported sediment. However, soil type almost has not affected ERoc in sediment but has a great impact on SOC loss through influencing sediment erosion pattern and the SOC content in original soils. The special formation processes of the soil on the Loess Plateau were considered the main reason. However, the rainfall intensity used in this study was relatively high. Thus, further studies with lower rainfall intensity should be conducted in the future.
